# Supplementary material for: GWAS links APOE to neuropsychiatric symptoms in mild cognitive impairment and dementia
Source: medRxiv. 2025 Feb 2:2025.01.31.25321498. Preprint. [Version 1] doi: 10.1101/2025.01.31.25321498 (PMC11838693; doi:10.1101/2025.01.31.25321498)
Supplement: Supplement 1 [file media-1.docx]

## Supplementary Figure 1. QQ plots and lambda estimates from GWAS p-values per NPS.
